# The Role of the Intestinal Microbiota in Nonalcoholic Steatohepatitis

**DOI:** 10.3389/fendo.2022.812610

**Published:** 2022-02-08

**Authors:** Hui Xiang, Dating Sun, Xin Liu, Zhi-Gang She, Yonghong Chen

**Affiliations:** ^1^Infectious Disease Department, Chongqing University Three Gorges Hospital, Chongqing, China; ^2^Department of Cardiology, Renmin Hospital of Wuhan University, Wuhan, China; ^3^Department of Cardiology, Wuhan NO.1 Hospital, Wuhan, China

**Keywords:** intestinal microbiota, nonalcoholic steatohepatitis, SCFAs, choline, inflammation, gut-liver axis

## Abstract

Nonalcoholic steatohepatitis (NASH) is a serious disease threatening public health, and its pathogenesis remains largely unclear. Recent scientific research has shown that intestinal microbiota and its metabolites have an important impact on the development of NASH. A balanced intestinal microbiota contributes to the maintenance of liver homeostasis, but when the intestinal microbiota is disequilibrated, it serves as a source of pathogens and molecules that lead to NASH. In this review, we mainly emphasize the key mechanisms by which the intestinal microbiota and its metabolites affect NASH. In addition, recent clinical trials and animal studies on the treatment of NASH by regulating the intestinal microbiota through prebiotics, probiotics, synbiotics and FMT have also been briefly elaborated. With the increasing understanding of interactions between the intestinal microbiota and liver, accurate and personalized detection and treatment methods for NASH are expected to be established.

## Introduction

Non‐alcohol fatty liver disease (NAFLD) has become the most common chronic liver disease worldwide (1), with a global prevalence of more than 25% (2). Nonalcoholic steatohepatitis (NASH), the progressive stage of NAFLD, is characterized by hepatic steatosis, inflammation, ballooning and fibrosis (3). NASH is the leading cause of liver-related mortality worldwide because of its tendency to develop into cirrhosis and hepatocellular carcinoma (HCC), as well as its impact on extrahepatic diseases, such as cardiovascular disease (CVD) and chronic kidney disease (CKD) (4, 5). The pathogenesis of NASH, however, remains extensively elusive, which is one of the main factors hampering the development of pharmaceutical strategies of NASH. In recent years, accumulating studies have indicated that the intestinal microbiota mediates the progression of NASH by affecting gut barrier permeability, hepatic lipid metabolism, inflammation and fibrosis (6).

Approximately 40 trillion microbes inhabit the human gut (7), which mainly includes bacteria, archaea, viruses and fungi (8). Intestinal microbiota is usually associated with metabolic diseases of the human host, such as diabetes, obesity, CVD, NAFLD and NASH (5). To date, multiple clinical and preclinical studies have demonstrated that individuals with NASH usually have compositional changes in their intestinal microbiota (9). Intestinal microbiota dysbiosis has been shown to accelerate the development and progression of NASH (10). On account of this, more and more scientific research attempts to inhibit or treat NASH by intervening with the intestinal microbiota, such as probiotics, prebiotics and synbiotics supplementation and fecal microbiota transplantation (FMT) (10). The mechanisms by which intestinal dysbiosis promotes NASH will be highlighted in the review. Moreover, studies on the treatment of NASH by intervening intestinal microbiota have also been elaborated accordingly.

## Composition of Intestinal Microbiota in NASH Patients

A clinical study by Zhu et al. showed significant increases of *Bacteroidetes*, *Proteobacteria*, *Enterobacteriaceae* and *Escherichia* and decrease of *Firmicutes* and *Bifidobacterium* in the NASH group compared with the healthy group (11). However, Mouzak et al. found that NASH was significantly associated with a lower proportion of *Bacteroides* (12). Del Chierico et al. demonstrated that compared with healthy controls, *Ruminococcus*, *Blautia* and *Dorea* increased in NASH patients, while *Oscillospira* reduced (13). In addition, the progression from nonalcoholic fatty liver (NAFL) to NASH is also accompanied by changes in intestinal microbiota, but the relevant studies are insufficient, currently. Del Chierico et al. found an increase in *Firmicutes* and a decrease in *Bacteroidetes*, *Proteobacteria* and *Actinobacteria* in NASH patients compared with NAFL (13). Similarly, Schwimmer et al. found that NAFLD patients had higher abundance of *Oscillibacter*, *Lactonifactor*, *Akkermansia* and *Enterococcus*, while NASH patients were accompanied by higher abundance of *Lactobacillus* and *Oribacterium* (14). Taken together, multiple clinical studies have shown that compositional changes of intestinal microbiota are common in patients with NAFLD/NASH, but such changes remain largely inconsistent and contradictory due to the heterogeneous of the relative distribution of intestinal microbiota (**Table 1**). Therefore, more studies are urgently needed to clarify the compositional changes of intestinal microbiota in each stage of NASH, even beginning from the healthy stage, and this has positive implications for future treatments of NASH by targeting individual intestinal microbiota.

**Table 1:** Composition of intestinal microbiota in NASH patients.

Models	Method	Main conclusions		Ref.
		Increased	Decreased	
NASH	16S rRNA pyrosequencing	Bacteroidetes, Proteobacteria, Enterobacteriaceae, Escherichia,	Firmicutes, Bifidobacterium	(11)
NASH	Quantitative real-time polymerase chain reaction	C. coccoides	Bacteroidetes	(12)
NASH	16S rRNA pyrosequencing	Ruminococcus, Blautia, Dorea	Oscillospira	(13)
NASH	16S rRNA gene sequencing	Collinsella	Ruminococcaceae	(15)
NASH	16S rRNA gene sequencing	Bacteroidetes, Proteobacteria, Enterobacteriaceae, Escherichia	Firmicutes, Bifidobacterium	(16)
NASH	16S rRNA gene sequencing	Lactobacillus	Bacteroides, Bifidobacterium	(17)
children with NAFLD	16S rRNA gene microarray	Gamma proteobacteria, Prevotella	–	(18)

## The Key Mechanisms of How the Intestinal Microbiota Affects NASH Progression

### The Impact of Intestinal Microbiota-Derived Short Chain Fatty Acids (SCFAs) on NASH

**Figure 1:**
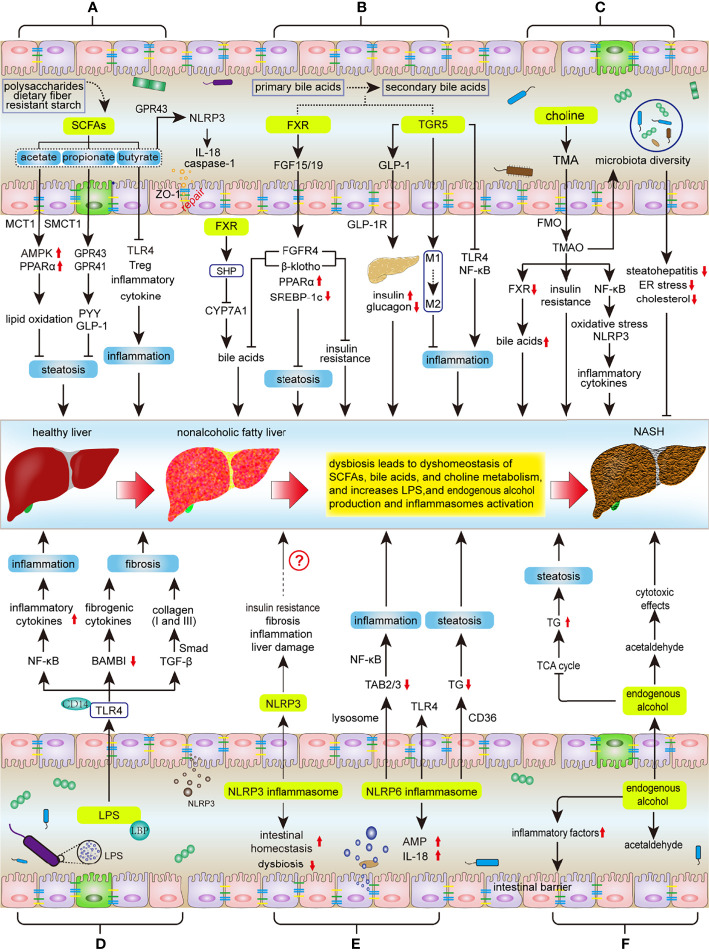
Key mechanisms involved in the regulation of intestinal microbiota during NASH progression. Intestinal dysbiosis results in disruption of intestinal SCFAs, bile acids, and choline metabolic homeostasis, as well as increases LPS and endogenous alcohol production and NLPR3/6 activation, subsequently affecting the progression of ANSH: **(A)** SCFAs inhibit hepatic steatosis, inflammation, and protect the integrity of the intestinal barrier. Dysbiosis decreases SCFA production, thereby promoting the NASH process. **(B)** The metabolism of bile acids is regulated by FXR and TGR5. FXR signaling suppresses hepatic steatosis and insulin resistance, as well as negative feedback inhibits bile acid synthesis; TGR5 can protect the liver from inflammation and insulin resistance. However, dysbiosis will reduce the activity of FXR and TGR5 signaling. **(C)** Intestinal microbiota metabolizes choline to TMAO, but the effect of TMAO on NASH is controversial. **(D)** LPS mainly affects the progress of NASH through LPS-TLR4 and NF-κB signaling pathways, including hepatic inflammation, fibrosis and liver injury. **(E)** Activation of NLRP3 in the liver promotes liver damage, but NLRP3 in the intestine maintains intestinal homeostasis and improves intestinal dysbiosis. NLRP6 inhibits NASH progression by inhibiting TLR4/NF-κB signaling and TG accumulation and promoting AMP and IL-18 secretion. **(F)** Intestinal microbiota increases the production of endogenous alcohol and promotes the progress of NASH. SCFAs, short chain fatty acids; MCT1, monocarboxylate transporter 1; SMCT1, sodium‐coupled monocarboxylate transporter 1; AMPK, AMP activated protein kinase; PPARα, Peroxisome proliferator-activated receptor α; GPR41/43, G protein-coupled receptor 41/43; IL-18, Interleukin 18; PYY, peptide YY; GLP1, Glucagon like peptide 1; TLR4, Toll-like Receptor 4; Treg, regulatory T; FXR, Farnesol X receptor; LRH-1, liver receptor homolog 1; CYP7A1, cholesterol 7a hydroxylated enzyme; FGF15/19, fibroblast growth factors 15/19; FGFR4, fibroblast growth factor receptor 4; SREBP-1c, sterol regulatory element-binding protein 1c; TGR5, Takeda G protein-coupled receptor 5; GLP-1R, GLP-1 receptor; NF-κB, nuclear factor-kappaB, TMA, trimethylamine; TMAO, trimethylamine-N-oxide; FMO, flavin monooxygenases; LPS, lipopolysaccharide; LBP, LPS binding protein; TGF-β, transforming growth factor-β; BAMBI, bone morphogenetic protein and active membrane-bound inhibitor; AMPs, antimicrobial peptides; NLRP3/6, nucleotide-binding domain, leucine-rich-repeat containing family, pyrin domain-containing 3/6; TAB2/3, TGF-β activated kinase 1 binding protein 2/3; TG, triglyceride; TCA, tricarboxylic acid.

Intestinal bacteria (such as *Ruminococcus*, *Anaerostipes*, *Bacteroidetes*, *Akkermansia muciniphila* and *Lachnospiraceae*) can degrade polysaccharides, dietary fiber and resistant starch into monosaccharides and SCFAs, and SCFAs of which mainly include acetate, propionate, butyrate (19, 20). Most SCFAs are utilized in the intestine (especially butyrate) to provide approximately 70% of the energy for intestinal epithelial cells (21). A small number of SCFAs enter the portal vein through monocarboxylate transporter 1 (MCT1) and sodium‐coupled monocarboxylate transporter 1 (SMCT1) receptors and subsequently infiltrate the liver (6). As a metabolic substrate and signaling molecule that regulates metabolism, SCFAs regulate hepatic metabolism through the gut -liver axis (**Figure 1**), which refers to the bilateral relationship between the gut and the liver through the portal system and biliary tract (22).

Animal studies have shown that SCFAs can activate AMP-activated protein kinase (AMPK) to accelerate fatty acid oxidation and inhibit hepatic lipogenesis, which leads to a decrease of hepatic lipid accumulation (23). For instance, Araújo et al. found that acetate produced by *E. coli* is absorbed by intestinal epithelial cells and metabolized into acetyl-CoA and AMP, as well as upregulates the AMPK/PGC-1α/PPARα pathway, subsequently promoting lipid oxidation (24). Acetate and propionate act as ligands to activate G protein-coupled receptors 41 (GPR41) and GPR43, leading to an increase of peptide YY (PYY) and glucagon-like peptide-1 (GLP-1) secretion, and thereby inhibiting energy intake (23). Butyrate increases hepatic GLP-1 receptor (GLP-1R) expression by inhibiting histone deacetylase-2 (HDAC-2) and activating AMPK (25). In adipocytes, SCFAs promote leptin expression by binding to GPR41 to promote glycometabolism and lipid and energy metabolism and to inhibit fatty acid synthesis (6).

In addition, SCFAs are beneficial to reducing hepatic inflammatory responses. SCFAs beneficially maintain the integrity of the intestinal barrier, which prevents intestinal toxins (e.g. LPS) from invading the liver (20). In colon epithelial cells, SCFAs activate the NLRP3 inflammasome by binding to GPR43, leading to caspase-1 activation and IL-18 release, thereby promoting epithelial repair (26). Butyrate can reduce endotoxin levels and hepatic inflammation by decreasing the expression of Toll-like Receptor 4 (TLR4) and CD14 and ameliorating dysbiosis and intestinal barrier function (increased claudin-1 and ZO-1 expression) (27, 28). Moreover, SCFAs reduces the generation of regulatory T (Treg) cells and inflammatory cytokines by inhibiting the activity of histone acetyltransferases, thereby reducing hepatic inflammatory responses (20). In methionine/choline-deficient diet (MCD)-induced NASH mouse model, Deng et al. demonstrated that SCFAs (sodium acetate) alleviated hepatic steatosis and inflammation by activating AMPK and inhibiting macrophage proinflammatory activation, respectively (23). In line with this result, Olaniyi et al. found that acetate ameliorates hepatic lipid dysregulation by inhibiting HDAC and enhancing insulin sensitivity (29). Therefore, SCFAs can be regarded as a novel and viable therapeutic compound for preventing and alleviating NASH.

### Intestinal Microbiota Regulates the Progression of NASH by Affecting the Metabolic Homeostasis of Bile Acids

As the prominent bacteria in the intestine, intestinal microbiota such as *Bifidobacterium*, *Bacteroides*, *Lactobacillus*,* Clostridium*, *Escherichia*, *Ruminococcus* and *Fusobacterium* play a pivotal role in the metabolism of bile acids by metabolizing primary bile acids into secondary bile acids (10, 30). The metabolism of bile acid plays an important role in liver homeostasis and regulates the NASH process by activating the bile acid receptors, including farnesoid X receptor (FXR) and membrane Takeda G protein-coupled receptor 5(TGR5) (**Figure 1**) (31). In the liver, FXR activates small heterodimer partner (SHP), which forms a polymeride with liver receptor homolog 1 (LRH-1), thereby suppressing the activity of cholesterol 7a hydroxylated enzyme (CYP7A1, a rate-limiting enzyme of bile acid synthesis) (32). In the gut, FXR promotes the expression of fibroblast growth factors FGF15/19, and further activates fibroblast growth factor receptor 4 (FGFR4) and β-klotho in hepatocytes, thereby inhibiting bile acid synthesis and reducing hepatic steatosis and insulin resistance (33, 34). In addition, FXR can also inhibit hepatic lipogenesis by activating PPARα and repressing the expression of SREBP-1c in SHP-79- and FGF 15/19-dependent manners (35).

In a western diet-induced NASH model, TGR5 activation stimulates the production of GLP-1 in intestinal endocrine cells, thereby increasing insulin secretion and decreasing glucagon synthesis by binding to GLP-1R in β-cells (36). TGR5 also suppresses inflammation by promoting the transformation of macrophages from the M1 to M2 phenotype and suppressing the activity of TLR4-NF-κB pathway (37, 38). Furthermore, TGR5 increases energy expenses by increasing the activity of thyroid hormone activating enzyme deiodinase 2, which increases conversion of T4 to T3 (39).

As reported, the abundance of bacteria that converts primary bile acids to secondary bile acids was decreased under NASH condition (10). Intestinal dysbiosis contributes to the disorders of bile acid metabolism, and results in an insufficient activation of FXR and TGR5, ultimately leading to lipogenesis and inflammation (10). Therefore, restoring the intestinal microbiota involved in bile acid metabolism in NASH patients is a promising treatment option. Previous studies have showed that the administration of probiotics altered the bile acid composition (40), and bile acid-based therapies, including hepatic FXR agonists, FGF15/19 analog, are considered for NASH therapy (31). A recent clinical trial (NCT02443116) showed that aldafermin, a FGF19 analog, suppressed bile acid synthesis, led to an enrichment of *Veillonella* and corresponding changes in serum bile acids in patients with NASH (41). Obeticholic acid (OCA), a FXR agonist, has shown effectiveness in altering the intestinal microbiota, and effectively improves hepatic histological characteristics in patients with NASH (42, 43).

### Intestinal Microbiota Regulates the NASH Process by Affecting Choline Metabolism

As an essential nutrient, choline was involved in liver lipid and cholesterol metabolism and signal transduction in bile acid enterohepatic circulation. Intestinal microbiota, such as *Desulfovibrio desulfuricans*, *E. coli*, *Clostridium*, *Anaerococcus hydrogenalis* and *Klebsiella pneumoniae*, can convert choline to trimethylamine (TMA) (44). TMA can be reabsorbed into the liver through the portal system, where it is metabolized by flavin monooxygenases (FMO) to generate trimethylamine-N-oxide (TMAO) (45). TMAO is correlated with the etiology and mortality of multiple diseases. Evidence indicates that higher serum level of TMAO was harmful to CVD and CKD (46), but controversial in NASH process (44, 47) (**Figure 1**).

Previous studies showed that TMAO aggravates liver steatosis by suppressing the activation of liver FXR signaling (48), upregulating glucose metabolism, and increasing insulin resistance (49). Furthermore, the increased TMAO levels induce the activation of the NF-κB pathway, promote oxidative stress and activate the NLRP3 inflammasome, thereby increasing the release of inflammatory cytokines such as IL-18 and IL-1β (46). However, recent evidence has demonstrated that TMAO modulated intestinal microbiota diversity, improved the histological alterations of steatohepatitis, alleviated hepatic endoplasmic reticulum (ER) stress, and inhibited the absorption of intestinal cholesterol in a high fat-high cholesterol (HFHC)-induced NASH mouse model (50). Despite this inconsistent observation, it is generally believed that decreasing choline levels and increasing toxic choline metabolites are crucial mechanisms by which the intestinal microbiota promotes NASH progression (51), which is the reason why MCD was considered a common dietary pattern for inducing NASH models.

### LPS Released by Intestinal Microbiota Aggravates the Progression of NASH

LPS, a component of gram-negative bacteria, has been identified as a major factor in NASH (45, 52). The association between LPS and intestinal dysbiosis has been well reported (45), the increased* Bilophila wadsworthia*, *Atopobium* spp., *Clostridium cocleatum* and decreased *Bifidobacterium*, *Bacteroides*, and *Eubacterium*, all leading to an increase of serum LPS concentrations (53).

LPS deteriorates NASH progression mainly by inducing the hepatic inflammatory response and fibrosis *via* LPS/TLR4 and NF-κB signaling pathways(**Figure 1**) in hepatocytes, hepatic stellate cells (HSCs) and Kupfer cells (54). LPS binds to LPS-binding protein (LBP) and then activates CD14-TLR4 to form a complex, subsequently promoting the release of inflammatory cytokines such as NF-κB (54). Carpino et al. found that LPS induces the activation of macrophages and platelets through the TLR4 pathway, thereby eliciting liver damage (52). LPS also promotes the expression of TGF-β, which induces the transcription of type I and III collagen *via* Smad-dependent pathways, thereby promoting hepatic fibrosis (55). In HSC cell line, TLR4 directly downregulates the bone morphogenetic protein and active membrane-bound inhibitor (BAMBI) to produce fibrogenic cytokines and activate TGF-β-mediated HSCs (56). Moreover, LPS directly induces oxidative stress, which is one of the most important pathological events in the development of NASH (54), targeting intestinal microbiota offers a therapy potential to reduce LPS concentrations and ameliorate NASH (57, 58).

### The Activation of Inflammasomes Triggered by the Intestinal Microbiota Affects the Development of NASH

Inflammasomes are multiprotein complexes assembled from cytoplasmic pattern recognition receptor (PRR), which mainly include NLRP1, NLRP3, NLRC4, and AIM2 (59). To date, NLRP3 and NLRP6 are considered to be closely related to microbe-induced NASH (**Figure 1**). Intestinal dysbiosis promotes the entry of PAMPs, DAMPs and LPS into the portal circulation through the impaired intestinal barrier, which leads to the activation of NLRP3 and inflammation in the liver *via* TLR4 signaling and Kupffer cells (60). Mridha et al. found that MCC950, a NLRP3 selective inhibitor, reduces hepatic inflammation and fibrosis in MCD-fed mice by decreasing the expression of pro-IL-1β, and normalizing caspase 1, IL-6, IL-1β, and MCP-1 levels in the liver (61). HSCs engulfed NLRP3 particles increase IL-1β secretion and α-smooth muscle actin (α-SMA) expression, thereby inducing hepatic fibrosis (62). Moreover, Dong et al. found that NLRP3 activation in HSCs of mice exacerbated the progression of NASH to hepatic fibrosis through the TLR4-NF-κB signaling pathway (63). NLRP3 instigates insulin resistance (64), and participates in the transition from NAFLD to NASH (65). However, NLRP3 inflammasome deficiency exacerbates gut-liver axis derangement, dysbiosis, steatohepatitis and liver damage in HFHC-fed Nlrp3^-/-^ mice (66). These studies suggest that NLRP3 may have different activities in different organs, thereby promoting liver damage and protecting intestinal permeability and preventing bacterial translocation (67).

NLRP6 is highly expressed in the small intestine and colon, especially in colonic goblet cells, myofibroblasts, and enterocytes (68). Intestinal microbiota induces NLRP6 signaling to produce IL-18 in mice, which is necessary for antimicrobial peptide (AMP) induction in the colonic mucosa (69). The deletion of NLRP6 alters the configuration of the intestinal microbiota, which leads to hepatic steatosis and inflammation *via* TLR4 signaling (70). In NASH mouse models, Huang et al. found that NLRP6 accelerates the degradation of TGF-β activated kinase 1 binding protein 2/3 (TAB2/3) through a lysosomal dependent pathway, thereby suppressing NF-κB mediated inflammatory responses (70). Also, NLRP6 inhibits hepatic TG accumulation by regulating CD36-mediated fatty acid uptake (70). Therefore, targeting the intestinal microbiota to regulate the activity of NLRP3 and NLRP6 is a promising therapeutic strategy to treat NASH.

### Endogenous Alcohol Produced by the Intestinal Microbiota Promotes NASH Progression

Although NASH is defined as absence of alcohol intake, endogenous alcohol, produced by intestinal microbiota such as high-alcohol-producing Klebsiella pneumoniae (HiAlc-*Kpn*), *Bacteroides*, *Bifidobacterium*, and *Escherichia*, is a non-negligible pathogenic factor in NASH progression (11, 71). It has been reported that patients with NASH have more bacteria associated with elevated blood alcohol levels (72). Zhu et al. found that compared with healthy controls, the serum alcohol concentration was strikingly elevated, as well as the remarkably increased abundance of *Escherichia* in NASH patients (11). Similarly, Yuan et al. found that HiAlc-*Kpn* aggravates hepatic steatosis, inflammation, mitochondrial dysfunction and liver injury by producing excessive ethanol (71). On the one hand, alcohol increases the expression of intestinal inflammatory factors and destroys the intestinal barrier, which is associated with small intestinal bacterial overgrowth, and aggravates intestinal dysbiosis (73) (**Figure 1**). On the other hand, endogenous alcohol inhibits the tricarboxylic acid(TCA) cycle, and aggravates hepatic triglyceride accumulation and deposition (74). Furthermore, the toxic intermediates of alcohol metabolism (acetaldehyde) and alcohol disorders the function of intestinal tight junction proteins (75). Therefore, reducing the production of endogenous alcohol by targeting alcohol-producing bacteria in the intestine is a promising direction for NASH treatment.

## Intestinal Microbiota as a Potential Therapeutic Strategy for NASH Treatment

To date, there are no Food and Drug Administration (FDA)-approved special drugs for NASH clinical treatment (76). Recently, an increasing number of clinical trials (**Table 2**) and animal experiments (**Table 3**) have attempted to prevent the development of NASH by targeting intestinal microbiota, including probiotics, prebiotics, synbiotics and FMT.

**Table 2:** Intestinal microbiota-targeted therapies of NASH-clinical trials.

Interventions	Samples and models	Main conclusions	NCT
**Probiotics**			
VSL#3	48 Obese children with NASH	VSL#3 improved NAFLD in children	NCT01650025
Multi-probiotic “Symbiter”	58 patients with NAFLD	Significantly decreased fatty liver index, serum AST, TNF-α and IL-6 levels	NCT04450875
Lactobacillus bulgaricus and Streptococcus thermophilus	30 patients with NAFLD	Improved hepatic aminotransferases levels	NCT02764047
Probiotics and metformin	64 patients with NASH	Improved hepatic aminotransferases, cholesterol, and TG contents	NCT02764047
Probiotic +omega-3 Fatty Acids	48 patients with NAFLD	Decreased fatty liver index, serum triglycerides, and total cholesterol	NCT03528707
Lepicol probiotic	20 patients with NASH	Reduced liver fat and AST levels	NCT00870012
**Prebiotics**			
OFS-enriched inulin	60 patients with NAFLD	Attenuated liver steatosis and fibrosis	NCT02568605
OFS	14 patients with NASH	Reduced histologically-confirmed hepatic steatosis	NCT03184376
**Synbiotics**			
Synbiotic supplement	50 patients with NAFLD	Significantly reduced hepatic steatosis and fibrosis, and improved HOMA-IR and insulin sensitivity	NCT02530138
OFS and Bifidobacterium animalis subsp. lactis BB-12	104 patients with NAFLD	Altered the fecal microbiome	NCT01680640
Synbiotic	52 patients with NAFLD	Significantly reduced AST, ALT, TNF-a and fibrosis score	NCT01791959

**Table 3:** Intestinal microbiota-targeted therapies of NASH-animal experiments.

Interventions	NASH models	Main conclusions and mechanisms	Ref.
**Probiotics**			
Blueberry juice and probiotics (BP)	HFD—induced NASH	BP prevents NASH development by inhibiting SREBP-1c/PNPLA-3 pathway *via* improving the activity of PPAR-α.	(77)
VSL#3	Western diet -induced NAFLD	VSL#3 normalized bile acid homoeostasis by changing the metabolic pathway of bile acids and activating the ileal G protein-coupled BA receptor 1 (GPBAR1) signaling.	(78)
Lactobacillus plantarum NA136	high-fat and fructose diet-induced NAFLD	L. plantarum NA136 improved IR, dysbiosis, and the expression of intestinal tight junction proteins (ZO-1, claudin-1 and occludin), and reduces NF-κB mediated inflammation.	(79)
Probiotics plus ARB	CDAA- induced NASH	Probiotics plus ARB inhibited the expression of liver TLR4 and LBP and improved the permeability of the intestinal barrier.	(80)
Lactobacillus plantarum NA136	high-fat diet and fructose -induced NAFLD	L. plantarum NA136 attenuated NAFLD by increasing the activation of AMPK/Nrf2 pathway, which will improve hepatic lipid metabolism and ameliorate oxidative stress.	(81)
Bifidobacteria and resveratrol	HFD-induced NAFLD	Significantly alleviated obesity and NAFLD.	(82)
**Prebiotics**			
Fructooligosaccharides (FOS)	MCD diet-induced NASH	FOS ameliorated hepatic inflammation by reducing the expression of TLR4 and CD14, and improved steatosis and tight junctions by regulating the production of SCFAs.	(83)
Isomalto-oligosaccharides (IMOs)	HFD-induced NAFLD	IMOs improved intestinal microbial abundance, systemic inflammation and endotoxemia.	(84)
FOS	monosodium glutamate (MSG) - induced NASH	FOS ameliorated steatohepatitis and chronic inflammation by increasing SCFA production and decreasing the M1 macrophage frequency.	(85)
**Synbiotics**			
L. paracasei B21060 based synbiotic	HFD-induced NAFLD	The synbiotic improved the permeability of the intestinal barrier, increased the expression of PPARα and FGF21, and reduced TLR2,4,9 mRNAs expression.	(86)
Synbiotic 2000^®^Forte (Synb)	high-fat choline deficient diet-induced NASH	Synb reduced serum levels of LPS and ameliorated hepatic fibrosis.	(87)
Bifidobacterium infantis and milk oligosaccharides	Western diet-induced NASH	B. infantis and MO increased TGR5-regulated signaling, and reduced bile acid synthesis by decreasing hepatic CYP7A1.	(88)
**FMT**			
8-week FMT intervention	HFD-induced NASH	FMT alleviated steatohepatitis by correcting microbiota disturbance and increasing the production of butyrate.	(89)
transfer the intestinal microbiota remodeled by pectin diet	HFD-induced NAFLD	FMT improved steatosis by increasing in the concentration of SCFAs.	(90)

Probiotics are living microorganisms that produce health benefits to the host, mainly including *Lactobacillus*, *Bifidobacterium* and *Streptococcus* (91). Both clinical and preclinical studies have congruously shown that probiotics dramatically ameliorate the histological spectrum of NASH (92, 93). For example, VSL#3 is a mixture of *Streptococcus*, *Thermophilus*, *Bifidobacterium* and *Lactobacillus* (94). The administration of VSL#3 significantly inhibited activation or production of JNK, NF-kB, α-SMA, metalloproteinases (MMP) and Cyclooxygenase 2 (COX-2), improved the intestinal permeability and alleviating oxidative stress, all of which were conductive to NASH therapy (94, 95).

Prebiotics, mainly including various oligofructoses (OFSs) or fructooligosaccharides (FOSs), refer to dietary supplements and indigestible food ingredients. Prebiotics improve host health by selectively stimulating the growth and activity of beneficial bacteria (96). Cani et al. found that prebiotics increased the production of proglucagon-derived peptide (GLP-2) in ob/ob mice, subsequently reducing plasma LPS levels and decreasing hepatic inflammatory and oxidative stress (97). Other studies have consistently shown that FOS attenuates hepatic lipid accumulation and steatohepatitis (84). In clinical trials, prebiotics have shown impressive efficacy in treating NASH, mainly by reducing serum alanine aminotransferase (ALT), aspartate aminotransferase (AST) levels and hepatic inflammation and increasing the abundance of *Faecalibacterium prausnitzii* and *Bifidobacterium* (98).

Synbiotics, the combination of probiotics and prebiotics, significantly inhibits NASH with liver histology improvement and inflammatory cytokines and endotoxin decrease (99). For instance, in a randomized clinical trial conducted by Ferolla et al. found that synbiotics supplementation effectively reduced hepatic steatosis and improved BMI and waist circumference (100). The mechanisms may be that synbiotics inhibited the subclinical pro-inflammatory signaling and reduced liver fat without altering the existing intestinal microbiota (100, 101).

FMT is an emerging and underexplored method to alternate the intestinal microbiota. FMT has been used in the treatment of a variety of gastrointestinal diseases, such as clostridioides difficile infections (102), diabetes (103) and irritable bowel syndrome (104). However, regrettably, there are no published clinical reports on the role of FMT in patients with NASH, but animal studies have shown that FMT treatment significantly improves the intestinal microbiota diversity and alleviates steatohepatitis (105–107). Zhou et al. found that FMT corrected the intestinal dysbiosis in HFD-fed mice and increased butyrate concentration and ZO-1 expression, thereby alleviating hepatic steatohepatitis (89). Vrieze et al. found that FMT from healthy donors to recipients with metabolic syndrome increased insulin sensitivity and butyric-producing microbiota of the recipient (108). Therefore, as a contemporary emerging therapeutic technology targeting the intestinal microbiota, FMT is undoubtedly regarded as a promising method for NASH treatment, but more clinical studies are urgently needed.

## Conclusion

Due to the emergence of 16S ribosomal RNA sequencing and metagenomics technologies, great progress has been made in research on the intestinal microbiota in recent years. The intestinal microbiota is related to a variety of human diseases mainly through metabolic pathways, such as regulating lipometabolism and glycometabolism. Studies focusing on the intestinal microbiota provide satisfactory opportunities for the pathogenesis and treatment of NASH. However, great challenges have to be borne. Many pending issues and challenges in this field need to be elaborate urgently. For example, the changes in the composition of the intestinal microbiota in NASH patients and healthy individuals remain largely elusive and controversial. The specific mechanisms of dysbiosis affecting the progress of NASH need to be further understood. In addition, many studies have been conducted only on rodent models. More clinical evidence and/or results obtained from nonhuman primates are needed to validate the results from experiments conducted on rodent models. There have been many clinical trials using prebiotics, probiotics and synbiotics to treat NASH, but these trials are relatively new strategies that lack specific mechanisms and large clinical trials with comparative endpoints. Therefore, intestinal microbiota analysis based on metagenomics may become a promising direction for the diagnosis and treatment of NASH in the future. Ultimately, intestinal microbial targeted precision medicine in the treatment of NASH, as well as early, accurate and non-invasive diagnostic and prevention methods for NASH are expected to be established.

## Author Contributions

YC and Z-GS contributed to conception and design of the study. HX wrote the first draft of the manuscript. DS and XL reviewed and edited the manuscript. All authors contributed to manuscript revision, read, and approved the submitted version.

## Conflict of Interest

The authors declare that the research was conducted in the absence of any commercial or financial relationships that could be construed as a potential conflict of interest.

## Publisher’s Note

All claims expressed in this article are solely those of the authors and do not necessarily represent those of their affiliated organizations, or those of the publisher, the editors and the reviewers. Any product that may be evaluated in this article, or claim that may be made by its manufacturer, is not guaranteed or endorsed by the publisher.
